# Harnessing technology for infectious disease response in conflict zones: Challenges, innovations, and policy implications

**DOI:** 10.1097/MD.0000000000038834

**Published:** 2024-07-12

**Authors:** Okechukwu Paul-Chima Ugwu, Esther Ugo Alum, Jovita Nnenna Ugwu, Val Hyginus Udoka Eze, Chinyere N Ugwu, Fabian C Ogenyi, Michael Ben Okon

**Affiliations:** a Department of Publication and Extension, Kampala International University, Uganda, Kampala, Uganda.

**Keywords:** conflict zones, epidemiological surveillance, infectious disease, security threats, technology interface

## Abstract

Epidemic outbreaks of infectious diseases in conflict zones are complex threats to public health and humanitarian activities that require creativity approaches of reducing their damage. This narrative review focuses on the technology intersection with infectious disease response in conflict zones, and complexity of healthcare infrastructure, population displacement, and security risks. This narrative review explores how conflict-related destruction is harmful towards healthcare systems and the impediments to disease surveillance and response activities. In this regards, the review also considered the contributions of technological innovations, such as the improvement of epidemiological surveillance, mobile health (mHealth) technologies, genomic sequencing, and surveillance technologies, in strengthening infectious disease management in conflict settings. Ethical issues related to data privacy, security and fairness are also covered. By advisement on policy that focuses on investment in surveillance systems, diagnostic capacity, capacity building, collaboration, and even ethical governance, stakeholders can leverage technology to enhance the response to infectious disease in conflict settings and, thus, protect the global health security. This review is full of information for researchers, policymakers, and practitioners who are dealing with the issues of infectious disease outbreaks in conflicts worn areas.

## 1. Introduction

Infectious diseases outbreaks in conflict areas are intersections of humanitarian crises and public health emergencies which are complex and require creative solutions.^[1]^ Such conflicts worsen existing vulnerabilities in healthcare infrastructure, interfere with surveillance and response activities, and make people more susceptible to the spread of disease.^[2]^ Solving the problems of infectious diseases in such environments demands a comprehensive interdisciplinary response linking technological innovations with classical public health and humanitarian approaches.^[3]^ The aim of this review is to focus on the cross-cutting issues concerning technology and infectious disease response in conflict zones, including the various challenges, the emerging innovative solutions, and the policy implications for future interventions. This review explores how the destructive effects of conflict on healthcare infrastructure results in reduced access to healthcare services and resources. Migrations result in increased pressure on healthcare systems, widening disparities, and disrupting disease surveillance and response activities.^[4]^ Rapid and efficient response to outbreaks of infectious diseases is important in order to stop the situation from escalating and curb the spread of diseases.^[5]^ Nevertheless, the threats to healthcare workers and humanitarian organizations in conflict areas are among the greatest obstacles to providing necessary health care services and responding to effectively.^[6]^ This review investigate the intricacies of protecting frontline responders when working in dynamic settings in order to deliver life-saving interventions. Innovations in epidemiological surveillance have transformed monitoring and management of diseases which provides with the actual disease dynamics and transmission patterns.^[7]^ In the same way, mobile health (mHealth) technologies have changed healthcare delivery through remote monitoring, telemedicine consultations, and individualized interventions.^[8]^ This paper also investigate how these innovations are being modified and applied in conflict zones, to improve surveillance capabilities and access to healthcare. Genomics sequencing tools have become a new weapon for fast pathogen identification and follow up, allowing personalized medicine and targeted treatment options.^[9]^ Meanwhile, issues associated with the standardization of protocols and ethical dilemmas in the area of data exchange as well as privacy are still relevant.^[10]^ This review consider the consequences of incorporating genomic sequencing into infectious disease response plans in conflict areas and the possibility of bettering patient outcomes. Surveillance technologies in the form of drones, and AI, are providing new ways of monitoring conflict trajectories and predicting outbreaks of diseases.^[11]^ Although technologies of this type promise to improve preparedness and response, some ethical concerns over surveillance and data protection need to be handled with caution.^[12]^ Lastly, this paper present policy suggestions on how technology should be used in the response strategies to infectious diseases in conflict areas with a focus on the need for surveillance systems, diagnostic tools, and capacity building. This review transparent governance systems and participation of stakeholders to facilitate responsible use of technology, protect the rights of individuals and make optimal resource allocations. With the purpose of providing guidance for future research agendas and informing decision-making to enhance public health outcomes in such complex environments, this review aims to explore the challenges, innovations, and policy implications of technology integration in the response to infectious disease in conflict zone.

## 2. Methods

This narrative review utilized a systematic approach to find and curate relevant literature on the relationship between technology and infectious disease responses in conflict zones.

### 2.1. Inclusion criteria

Studies that are published in high-impact factor peer-reviewed journals.

The articles’ content includes infectious disease outbreaks in conflict zones that comprise, but are not limited to, war zones, areas of political instability, and regions affected by armed conflict.

Study regarding technology incorporated into infectious disease surveillance, diagnosis, treatment, and response strategies.

Researches in the field of healthcare infrastructure and healthcare service access as affected by war induced destruction.

Literature on health care delivery and response intervention challenges for health care workers and humanitarian organizations in conflict areas.

The articles that review innovative technological approaches, particularly, the improvements in epidemiological surveillance, mHealth technologies, genomic sequencing, and surveillance technologies.

### 2.2. Exclusion criteria

Non-English published studies.

Non-peer-reviewed literature that is conference abstracts, editorials, and opinion pieces.

Articles that concentrate only on noncommunicable diseases at any given conflict zone.

Research that is not focused on implementation of technology in response to infectious disease in conflict areas.

Studies that are not related to the context of this review such as those that only target the technology developments in unrelated infectious disease control and conflict settings.

*Search strategy*: A systematic search strategy was used to find the relevant study though electronic databases such as PubMed, Web of Science, and Scopus. Combinations of Key words and Medical Subject Headings terms associated with infectious disease outbreaks, conflict zones, and technology integration were applied in different permutations in order to optimize associated literature retrieval. The search strategy was not performed in one go; the search results were initially screened on title, then abstract, and full-text eligibility criteria.

*Data extraction and synthesis*: The data extraction was conducted in a systematic manner in order to capture the major findings, methodologies, and implications from the chosen studies. Extracted information comprised study objectives, methodologies, findings, and implications for technology integration in infectious disease response strategies in conflict zones. Thematic synthesis of the findings was done, whereby common themes, challenges, and innovative solutions found across the reviewed literature were identified.

*Quality assessment*: Owing to the narrative structure of this review, no formal quality appraisal tools were used. Nonetheless, attempts were done to give a critical assessment on the validity of the selected studies considering their method and diligence as well as their relevance to the review objectives.

*Ethical considerations*: This review was compliant with the ethical principles for human subjects research and privacy of data. Every piece of information in the review was taken from publicly available sources, and there was no personal data revealed. Moreover, ethical aspects in the respect to correct usage of technology in conflict environment were looked through and discussed in terms of the review.

*Limitations*: All attempts to do a complete review notwithstanding, it may be that some studies might have been missed because of limitations in search strategy or database coverage. Also, non-English literature was not included that may have introduced language bias. In addition, the narrative style of this review prevents from quantitatively evaluating strength of evidence or performing a kind of formal quality assessment of included studies.

### 2.3. Overview of unique challenges posed by infectious disease outbreaks in conflict zones

Infection disease outbreaks in the conflict zones are a multi-faceted and intricate issue that unites the destruction of war with public health emergencies.^[13]^ This review gives a summary of the special problems caused by such outbreaks, such as compromised health infrastructure, refugee-helper services, limited access to healthcare, collapse of social as well as governmental structures as well as increase in the risk of disease transmission. The challenges to comprehend and overcome are necessary to develop responses and mitigation strategies in war-torn areas.^[14]^ Conflict areas are characteristically susceptible to epidemic outbreaks because of poor health systems, population displacements, and loss of basic services.^[15]^ If infectious diseases are combined with the condition of conflict, their effects become worse, more morbidity, mortality and social-economic instability occur.^[16]^ This review seeks to investigate the particular difficulties that one face in controlling infectious disease outbreaks during the period of conflict, and its impact public health interventions and humanitarian efforts. Health care infrastructure in conflict areas is usually highly compromised, in that hospitals are damaged, medical supplies are in short supply, and there are few trained health workers.^[17]^ This deficiency substantially limits the potential to recognize, diagnose, and manage infectious diseases properly.^[18]^ Secondly, the loss of medical centers reduces the opportunities for utilizing the services, increasing the load of diseases among the affected people.^[19]^ Refugee camps or informal settlements remain overcrowded because of the mass migration of populations caused by conflict.^[20]^ Such conditions are an excellent opportunity for rapid dissemination of infectious diseases, aided by close confinement, lack of proper sanitation, and limited availability of clean water.^[21]^ Victims of displacement are usually very unprotected, having no healthcare and being at great threat of malnutrition and communicable diseases.^[22]^ In conflict areas, obstacles which block the access to health care services are based on security issues, bureaucratic barriers, and physical barriers like checkpoints or roadblocks.^[23]^ Humanitarian organizations find it difficult to arrange relief and medical care for the affected groups, thus widening the gap between the rich and the poor, while making it easier for the outbreaks of the diseases to be left undiagnosed and untreated.^[24]^ Challenges in disease surveillance, prevention, and control of diseases occur due to the failure of social and governmental systems in conflict regions.^[25]^ Poor governance, insecurity and displacement interrupt public health programs and hinder the application of preventive measures including vaccination campaigns and vector control.^[26]^ Moreover, mistrust of institutions leads to misinformation and poor response to community disease control activities.^[27]^ Situation of conflicts allow for the development of infectious diseases which may be brought about by overcrowding, malnutrition and poor sanitation.^[28]^ Disruption of health services, immigration and emigration among a certain population facilitates rapid spread of pathogens that could cause local or wide spread outbreaks.^[29]^ In addition, the utilization of arms, migration of wild animals, and ruination of infrastructure could facilitate the creation of new disease carriers or worsen existing dangers to health.^[30]^ Disease outbreaks in areas of conflicts are challenging and compounded by the multi-vulnerabilities of armed conflicts and public health emergencies.^[30]^ Solving these problems need a holistic approach and merges the humanitarian support, health actions, and conflict resolution initiatives.^[31]^ Reinforcement of health systems, improvement in disease surveillance, and provision of equitable healthcare access are required to reduce the impact of infectious diseases in conflict areas.^[32]^ Collaboration of governments, humanitarian organizations, and local communities is crucial in tackling the unique challenges of infectious disease outbreaks in conflict zones, ensuring the health and life quality of the affected people as shown in Table 1.^[33]^

**Table 1:** Challenges posed by infectious disease outbreaks in conflict zones.

S/N	Challenges	Description
1	Overcrowded Refugee Camps	Conflict affects the social infrastructure and displaces the vulnerable population to congested camps or informal settlements where disease can spread quickly through contact with other people, infections are easily contracted and potentially deadly illness like cholera may ensue due to poor hygiene and lack of adequate clean water to promote hygiene.
2	Vulnerability of Displaced Populations	Displaced persons are commonly out of reach of appropriate health care and their existing health conditions at high risk from both nutritional and infectious illnesses; correspondingly, their living status is fragile due to displacement.
3	Challenges in Humanitarian Assistance	Security concerns and access challenges limit these groups in their abilities to deliver aid and healthcare; outbreaks go uncontrolled and unchecked, worsening health inequity.
4	Disrupted Disease Surveillance and Control	Public health is affected negatively by the breakdown of the social and government systems which loses their ability to carry out disease preventive measures like vaccination, vector control, disease reporting, prevention, and control.
5	Mistrust and Misinformation	Due to lack of trust in institutions, there is misinformation, and hence a poor community response to disease control activities, making it even harder to contain the spread of diseases.
6	Factors Promoting Disease Spread	Population pressure, poor diet, inadequate hygiene, and utilization of force, movement of wildlife, and disruption of facilities play a key role in disease outbreaks and new diseases.
7	Compounded Vulnerabilities	Combining armed conflicts with public health threats forms a complex problem that should be solved with the help of integrated approach.
8	Holistic Approach Needed	Humanitarian, health and peace efforts need to be aligned in a complex interdisciplinary framework that targets system health strengthening, better surveillance for diseases and equitable health facility utilization.
9	Collaboration	Government agencies both in developed nations and the third world countries, humanitarian organizations and locals should all come up with the best measures to come up with viable solutions to counter those hurdles and enhance the wellbeing of the citizens of the affected countries.

### 2.4. Importance of timely and effective response to prevent escalation and mitigate the spread of diseases

Epidemics of infectious diseases, triggered by known pathogens or new viruses, can occur in a matter of days or weeks, spreading within and across borders, thereby resulting in substantial morbidity, mortality, and socioeconomic disruption.^[34]^ Rapid and efficient reactions are required to control outbreaks at the point of their occurrence and avoid the amplification of these cases into bigger scale epidemics or pandemics.^[35]^ This review discusses the crucial aspects of the rapid response mechanisms in disease control and prevention of their spread. The timeliness of response is critical in breaking the spread of diseases that are caused by infectious agents.^[36]^ Early identification, diagnosis, and intervention can reduce the rate of transmission and lessen the impact of outbreaks.^[37]^ Quick response makes it possible to implement containment measures like quarantine, isolation, contact tracing, and vaccination that are essential in preventing further spread.^[38]^ Late responses lead to exponential cases explosion, collapse of health care system, and makes the situation worse.^[39]^ Effective response strategies involve collaboration across a number of disciplines and sectors including public health authorities, healthcare providers, researchers, policymakers, and communities.^[39]^ Strong surveillance systems ensure early detection of outbreaks and threats in emergence.^[38]^ Early warning systems combined with data analysis and modeling give knowledge about disease dynamics and help in planning proactive responses.^[40]^ Early case identification, isolation and treatment requires access to fast and reliable diagnostic tests.^[40]^ Developments in molecular diagnostics, point-of-care tests, and surveillance technologies, increase capability to detect and contain pathogens in a timely fashion.^[41]^ Non-pharmaceutical interventions such as social distancing, wearing of masks, hand hygiene, and travel bans can reduce the transmission rates as well as the spread of disease especially when there are no specific therapeutics or vaccines.^[42]^ Vaccines are very important in the prevention of infectious diseases as well as in reducing their burden on people.^[43]^ Vaccines deployed on time with effective immunization campaigns can improve herd immunity and restrict the spread of pathogens.^[43]^ The transparency and timeliness of communication with public are vital for building trust, encouraging compliance with preventive measures and eliminating myths.^[42]^ The effective risk communication strategies prevent fear, panic, and stigmatization caused by disease outbreaks.^[40]^ Resource limitations, which are usually associated with funding, personnel, and infrastructure, can slow the expansion of response activities, especially in low-resource contexts.^[43]^ Decentralization of efforts and failure of coordination among actors both local and national levels can sabotage responses and create surveillance, communication, and intervention gaps.^[44]^ Prevention and public health behaviors are influenced by social and economic disparities, cultural practices, and political tendencies thus making disease control difficult.^[45]^ The complex and dynamic nature of infectious diseases, which are often characterized by the emergence of novel pathogens and the ever-increasing antimicrobial resistance, makes it difficult to predict and respond to outbreaks.^[40]^ The timely and efficient response is the key preventive measure against the escalation and the tool of the distribution control of infectious diseases.^[41]^ By placing emphasis on surveillance, rapid diagnostics, public health interventions, vaccination campaigns, and communication strategies, all types of stakeholders can improve readiness and resistance to outbreaks.^[46]^ Overcoming such challenges as resource limitations, coordination problems, and socioeconomic considerations is crucial for improving response ability and protecting global health security.^[47]^ Joint action across sectors and borders is key to tackling the changing threat scenarios and in protecting the vulnerable people from the wrath of infections.^[48]^

### 2.5. Impact of conflict-related destruction on healthcare infrastructure

In conflict zones, destruction is not limited to physical violence but also sabotage that has been carried on critical infrastructure including healthcare facilities and resources.^[49]^ The further disruption of health services in these areas aggravates the plight of the population already affected by the consequences of war.^[50]^ However, conflict effects are not limited to the immediate destruction of medical facilities while they also raze other critical infrastructure such as roads, water supply systems, and power grids.^[51]^ Hence, most of the existing healthcare facilities are also compromised, with limited resources and failing to live up to the increasing need for medicare.^[52]^ In addition, the movement of populations also causes the overload of already overstretched healthcare systems, resulting in overcrowding and depletion of resources.^[52]^ Restricted availability of healthcare facilities as a result of destruction caused by the conflict results in various problems for the people concerned.^[49]^ Geographical constraints and disruption of transport systems deny the patients’ access to the healthcare services, mainly, in the remote areas.^[50]^ Furthermore, financial limitations worsen inequalities in healthcare access, and poor communities are the ones that suffer most.^[51]^ In addition, healthcare infrastructure breakdown causes quality of care available to deteriorate, posing adverse health risks to affected population.^[52]^

The destruction associated with conflicts often brings about acute inadequacies of medical supplies, machinery, and human resource, thereby, obstructing delivery of vital healthcare services.^[51]^ The burning of pharmaceutical warehouses and manufacturing plants fosters in supply chains and aggravates shortages of life-saving medications.^[53]^ In addition, the outflow of healthcare providers from conflict areas diminishes the already scarce number of skilled personnel, thereby, more undermining the ability of healthcare systems to adequately address the needs of affected people.^[53]^ Solving the associated challenges of limited health infrastructure access in conflict-affected areas requires a multi-faceted approach that includes humanitarian aid, infrastructure reconstruction, and the development of capacity building.^[54]^ The restoration and fortification of healthcare infrastructure is very critical investment with a view to strengthening resilience across future conflicts and disasters.^[55]^ Besides, initiatives aimed at promoting the fair allocation of medical resources and personnel, especially in the marginalized areas, are a must for the universal access to healthcare.^[56]^ Additionally, it is important that close relationship is built between the local actors, international organizations, and humanitarian actors to help in coordinating efforts towards maximum impact in conflict-affected regions.^[57]^ The destruction caused by war to the health infrastructure poses a significant challenge to access to universal healthcare in such areas. Comprehensive initiatives must be employed to deal with the complex problems of limited access to health care resources including infrastructural renovation, resource mobilization, and capacity-building programs.^[58]^ Focusing on the resilience and sustainability of health systems in conflict areas stakeholders would be able to reduce the effects of conflict destruction on healthcare access and also improve the health situation of the populations affected.^[58]^

### 2.6. Displacement of populations and breakdown of community structures hinder surveillance and response efforts

The population dislocation has become a widespread global phenomenon due to various reasons such as conflict, natural disasters and the economic factors.^[59]^ At the same time, disintegration of community structures destroys the texture of social solidarity and support networks.^[60]^ These occurrences have important public health implications, especially regarding disease surveillance and response.^[59]^ Appreciating the linkages between population displacement, community structure disintegration, and the effect of these conditions on the performance of public health interventions is very critical in the development of appropriate strategies for managing the associated risks.^[60]^ Population displacement makes surveillance difficult by scattering people over various locations, hence, broken data collection and partial epidemiological profiles.^[61]^ Furthermore, the displaced populations may be made not to have health care services and be at a higher risk of infectious diseases, which make surveillance be even more difficult.^[62]^ The core of community structures breakdown causing the interferences to traditional modes of information transfer and distracts the systems of surveillance designed to be implemented through community participation.^[62]^ However, the disease outbreaks are detected and monitored later, which leads to the delay in the response measures, and thus, the disease is more likely to spread.^[63]^ Destroying of community structures hinders the response mechanisms through lack of faith in the authorities and blocking the communication of important health information^.[64]^ Poor communication pathways, as well as cultural barriers, inhibit the provision of healthcare services to the displaced populations, therefore increasing the danger of disease transmission.^[59]^ Additionally, displaced people influx into host communities overloads the established health care infrastructure and resources, therefore, complicating response efforts.^[60]^ The politicization of humanitarian aid in situations of conflict or instability can delay the provision of necessary services, which can worsen public health crises.^[61]^ The solution to the problems created by population displacement and breakdown in community structure is a combination of actions enumerated over the surveillance enhancement, community engagement, and resource mobilization.^[62]^ The use of innovative technologies such as mobile health applications and geospatial mapping has the potential to strengthen epidemiological surveillance, enabling real-time data collection and analysis in widely dispersed populations.^[63]^ Participating with local community leaders and stakeholders is critical for gaining trust, promoting interaction, and adapting response interventions to the unique needs of affected populations.^[64]^ In addition, resilient healthcare systems that respond well to changes in population dynamics is a critical investment in order to mitigate the impact of displacement on public health.^[65]^ The movement of populations and disorganization of community systems are major challenges to surveillance and response activities in public health.^[66]^ Overcoming these challenges involves an integrated approach that combines technological advancement, community participation, and strengthening the health system.^[67]^ Understanding the intricate relationship between population dynamics and public health outcomes enables the policy makers and other practitioners to come up with strategies that can counter the effects of displacement on surveillance and response systems, thus protecting the health and welfare of those vulnerable displaced populations.^[68]^

### 2.7. Security challenges of healthcare workers and humanitarian agencies in conflict areas

**Figure 1.**
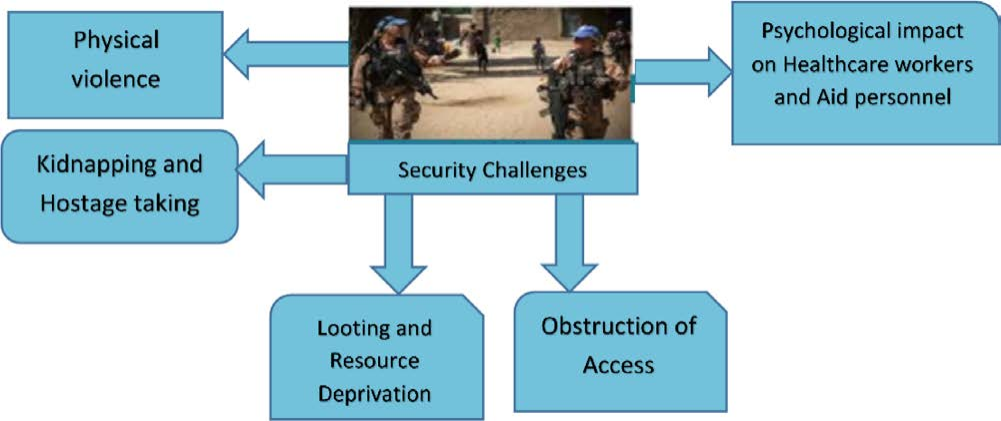
Security challenges of healthcare workers and humanitarian agencies in conflict areas.

In conflict zones, healthcare workers and aid organizations face substantial challenges as the delivery of critical services is frequently hampered by prevailing instability as shown in Figure 1 below. In such unstable environments, people and organizations dedicated to reducing the suffering and promoting health confront numerous security threats that compromise their safety, well-being and ability to provide help.^[69]^ This review explores the security environment experienced by health workers and organizations while working in the conflict areas, emphasizing the peculiarities of their operational environment and the necessity to devise appropriate risk mitigation strategies.^[69]^

### 2.8. Security risks

*Physical violence*: Violent incidents against healthcare workers and aid workers include direct physical attacks, such as targeted attacks, shootings, bombings, and assaults.^[70]^ These occurrences are a threat to lives of individuals and also disrupt the provision of essential healthcare services to the weak.^[71]^

*Kidnapping and hostage-taking*: Kidnapping of healthcare workers and aid workers in conflict zones is a continual risk due to diverse reasons such as ransom demands, political leverage, and ideological campaigns^.[72]^ The kidnaping of staff does not only cause immediate damage, but it also creates fear and uncertainty among humanitarian communities^.[73]^

*Looting and resource deprivation*: Looting of medical facilities, equipment and supplies in the course of conflict deprives healthcare workers of essential resources necessary for successful provision of care.^[70]^ In addition, looting tends to destroy the trust between humanitarian actors and local communities stirring up tensions and hampering humanitarian access.^[71]^

*Obstruction of access*: Bureaucratic impediments, checkpoints, roadblocks, and denials of entry by armed groups continuously impede the access of healthcare workers and aid organizations to those in need.^[69]^ These obstacles render humanitarian aid less effective and worsen the humanitarian crisis in conflict-affected zones.^[70]^

*Psychological impact on healthcare workers and aid personnel*: Working in conflict areas inflicts severe psychological damage to health workers and aid workers expressed through the symptoms of stress, anxiety, depression and post-traumatic stress disorder.^[71]^ Seeing violence, being exposed to personal threats, and dilemmas arising in moral grounds are the factors that have the highest impact on psychological distress among first responders.^[72]^ The combined effect of these stressors puts in risk the personal welfare of particular workers as well as weakens the ability of humanitarian operations to work effectively.^[73]^

### 2.9. Mitigating security risks and enhancing protection measures

*Security training and preparedness*: General security training programs should be provided to the healthcare workers and the humanitarian aid personnel in the conflict zones to acquire the skill of risk assessment, conflict, and personnel safety.^[74]^

*Community engagement and negotiation*: Creating a good relationship with both the local communities and the armed groups is very crucial in the security day to day operation and the access for the humanitarian intervention.^[75]^ Discussion, negotiation, and arbitration would assist in relieving tension and in fostering comprehension.^[75]^

*Operational adaptations*: Operational adaptations measures like mobile clinics, remote healthcare delivery, and decentralized aid distribution points can alleviate risks related to static healthcare facilities and supply chains.^[75]^

*International advocacy and diplomacy*: At the international level advocacy efforts and diplomatic mediation should also be stressed in order to guarantee that healthcare workers and humanitarian organizations would be safe at conflict zones, which should be followed by adhering to humanitarian principles and international humanitarian law.^[74]^

**Figure 2.**
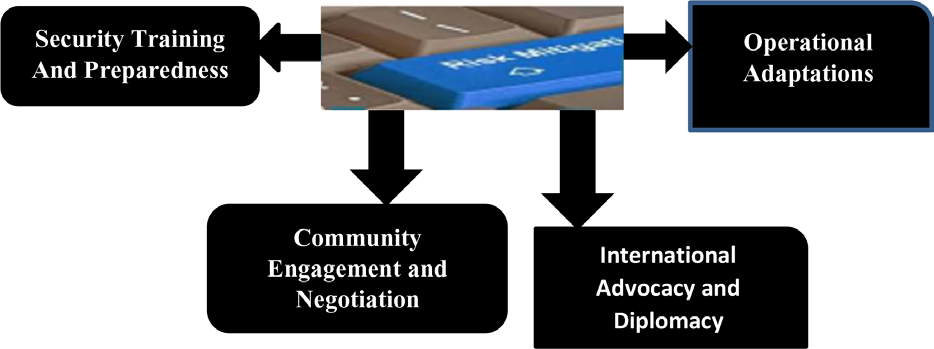
Mitigating security risks and enhancing protection measures.

The threats posed to health workers and humanitarian organizations by security in conflict-affected areas are multifaceted and go beyond denial of critical services and supplies in such areas.^[75]^ All these problems call for a holistic solution that involves the formulation of security training, participation of the communities, changes in policies as well as global advocacy as shown in Figure 2. The emphasis on the safety of the frontline responders and the reinforcement of security measures will increase the resilience and effectiveness of humanitarian operations in some of the most volatile area across the world.

### 2.10. Advancements in epidemiological surveillance

**Figure 3.**
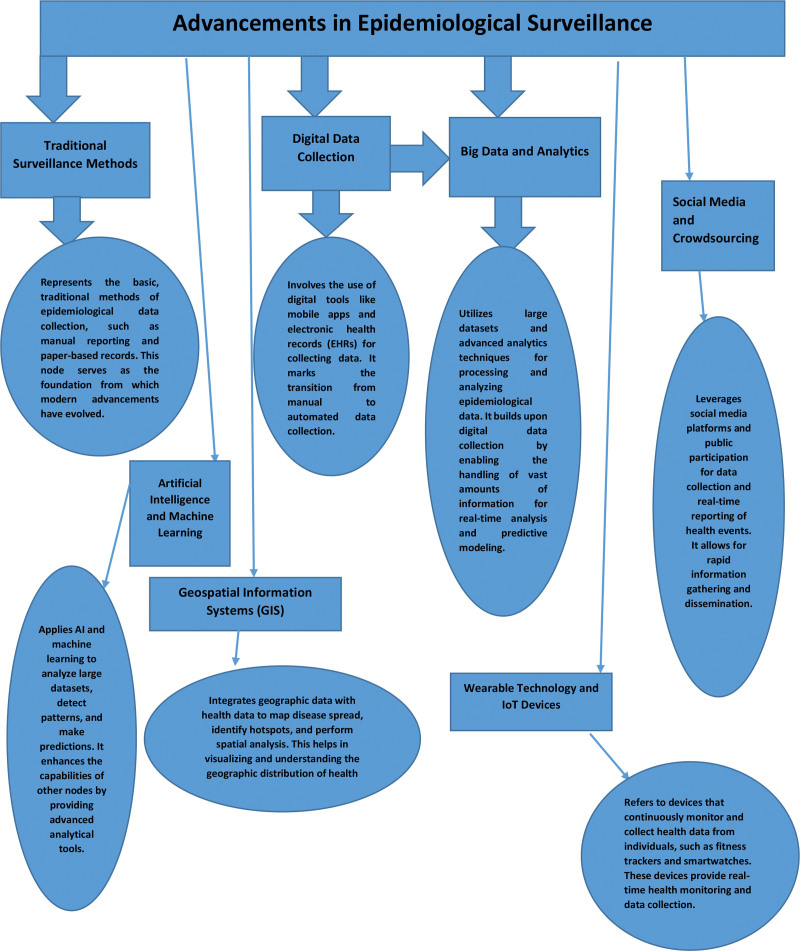
Advancements in epidemiological surveillance.

Surveillance epidemiology is a process that encompasses organized collection, analysis, interpretation, and distribution of health-related data to facilitate disease surveillance and control.^[76]^ In the past, surveillance was mainly based on the use of manual data collection methods that proved to be quite slow, labor intensive, and prone to errors^.[77]^ On the other hand, technological innovations of late enable new approaches to speed, adequacy, and extent of the surveillance as shown in Figure 3.

*Satellite imagery*: Satellite imagery has become an important instrument of epidemiological surveillance to collect information on environmental variables, demography, and vectors of disease^.[78]^ The high-detail satellite pictures allow for the tracking of land use, vegetation patterns, and water bodies—key disease transmission indicators. For instance, satellite images have been used to monitor the spread of vector-borne diseases like malaria and dengue fever by capturing the location of the mosquito breeding sites^.[79]^ Furthermore, satellite information can be used in combination with geographic information systems to produce predictive models of disease events and areas at high risk suitable for targeted interventions^.[70]^

*Drones*: Drones, which are unmanned aerial vehicles (UAVs), provide an economical and flexible way for spatial data gathering in inaccessible or dangerous areas.^[73]^ Drones can be utilized in a variety of applications in health surveillance, which include overhead surveys, protestor control, and sampling, among others^.[72]^ With drones, thermal imaging cameras can be used to trace the heat signatures of animal reservoirs of zoonotic diseases, while the fixed-wing drones can be used for more efficient coverage of large geographic areas than traditional ground-based surveys.^[70]^ Besides, they are capable of being quickly deployed in case of an emergency, ensuring the real-time information fueling the decisions in disease outbreaks or natural calamities.^[74]^

*Artificial intelligence (AI*): Epidemiological surveillance is now being applied in AI algorithms to facilitate automation of data analysis, pattern detection, and prediction of disease trends^.[80]^ Machine learning methodologies like neural networks and deep learning are capable to handle large amount of complex data coming from various sources including electronic health records, social media, and sensor networks.^[81]^ Through real-time analysis of these data streams, AI systems can detect atypical patterns of symptoms or clusters that would suggest the occurrence of a new disease or pandemic.^[81]^ In addition, predictive models powered by AI enable public health bodies to distribute resources strategically and timely conduct focused interventions to control the spread of infectious diseases^.[82]^

*Challenges and future directions*: Although there are numerous advantages of new technologies for epidemiological surveillance, there are several challenges that should be tackled.^[76]^ These comprise concerns on data privacy, data reconciliation, technical capability, and the regulatory framework. In addition, the fair sharing of technology resources and participation of local communities are essential for the validity and continuity of the surveillance systems.^[78]^ In the future, research should emphasize interdisciplinary partnerships, capacity building activities and tools and platforms development that will simplify the integration of satellite images, drones, and AI into surveillance activities.^[79]^

Thus, the combination of satellite imagery, drones, and AI, if implemented properly, can change the way of epidemiological surveillance forming it the exact and authentic bases for disease prevention and control.^[81]^ Utilizing these developing technologies, public health authorities can strengthen their capability to recognize, track, and contain threats posed by infectious diseases, which will in turn save lives and protect public health.^[82]^

### 2.11. Advancements in mHealth technologies for real-time data collection and transmission

**Figure 4.**
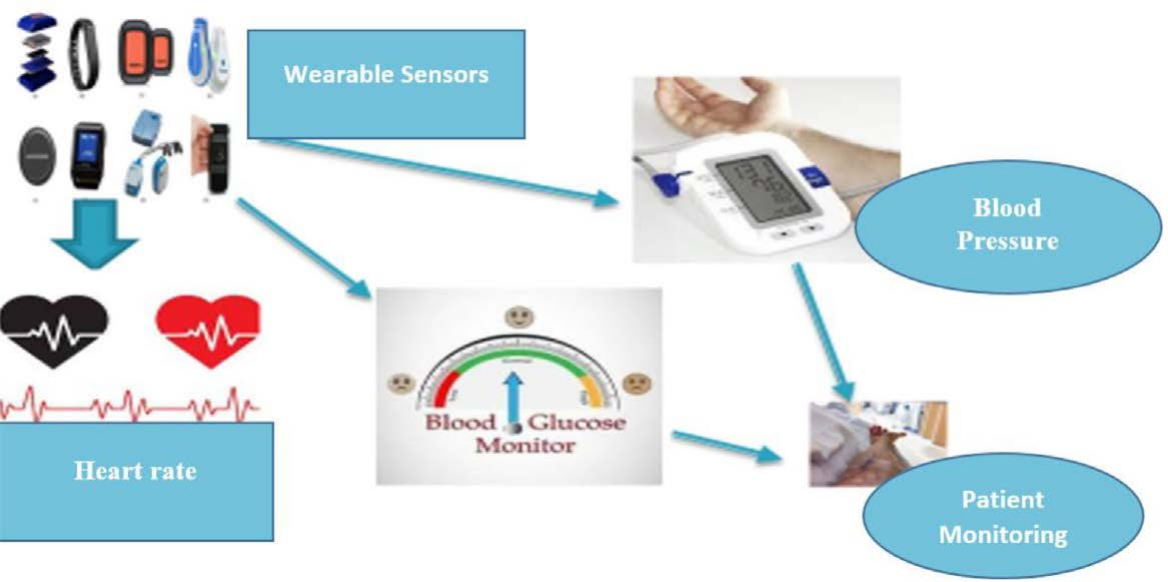
Wearable sensor technology revolutionizes real-time health monitoring.

MHealth technologies as a whole include various devices, applications, and platforms that are intended to enhance healthcare delivery, especially in remote or resource-limited environments as shown in Figure 4. Data collection and transmission in real time is one of the most important elements of mHealth systems which allows for continuous monitoring, immediate detection of health problems, and prompt interventions^.[83]^ This review takes a look into the development of mHealth technologies and how they transpire the face of healthcare.

*Wearable sensors*: The wearable sensors have evolved as tools for dynamic health monitoring. These devices, integrated in clothes, wristbands, or patches, gather physiological data like heart rate, activity level, and sleeping behavior^.[70]^ Modern sensors are also capable of detecting such vital signs like blood pressure, glucose levels, and oxygen saturation^.[71]^ Linking with smart phones facilitates smooth transmission of data to health care providers, leading to the remote monitoring of patients suffering from chronic diseases and early detection of anomalies^.[72]^

*Smartphone applications*: Applications, which are part of smartphone, do a great job in enabling the collection and transmission of real time data.^[74]^ Diet and exercise tracking features delivered in health and fitness apps help users maintain their diet, exercise, and medication adherence records, thus offering the valuable understanding of their health behaviors.^[75]^ Besides, specific applications for chronic disease management, mental health help, and medication notifications give the patients the ability to manage their health.^[50]^ By means of a secure connectivity, these applications provide access for the healthcare provider to the data generated by the patient eliminating any need of physical presence, enabling immediate personalized interventions leading to significant treatment outcomes.^[51]^

*Cloud computing*: Cloud computing technology is a base that allows stores and processes large amounts of health data that comes from mHealth devices and applications.^[52]^ The cloud-based platforms are scalable, accessible and data safe and thus provide real-time data management and analysis to the healthcare providers. In addition, cloud-based Electronic Health Record systems support complete integration with mHealth solutions, which improves care coordination and communication of health care teams.

*Wireless communication*: Wireless communication technologies, for example: Bluetooth, Wi-Fi, cellular networks, facilitate continuous data transfer among mHealth devices, smartphones, and cloud servers. Such technologies guarantee uninterrupted connection, even in far, and rural zones characterized by weak infrastructure.^[54]^ Further, the developments in wireless protocols and standards improve data security and inter-operability which in turns supports the wide use of mHealth solutions in healthcare settings.^[55]^

*Impact on healthcare delivery*: Integration of mHealth technologies for real-time data capture and transmission has revolutionized healthcare delivery through the remote monitoring, telemedicine consultations, and personalized interventions^.[55]^ These technologies provide better patient care, particularly in the underserved populations, which leads to cost reduction and improved patients’ engagement and satisfaction^.[56]^ Further, real-time data analytics enable healthcare professionals to make evidence-based decisions, improve treatment protocols, and predict health outcomes with greater precision^.[57]^

*Challenges and future directions*: Nonetheless, there remain the challenges associated with mHealth technologies such as worries about data privacy, regulatory issues, inter-operability problems, and digital divide^.[60]^ Addressing these challenges requires collaboration among the stakeholders that is health care providers, technology developers, policy makers, and researchers. The new areas in mHealth research include the implementation of AI, machine learning, and predictive analytics as well as the personalized and proactive healthcare interventions^.[66]^

**Figure 5.**
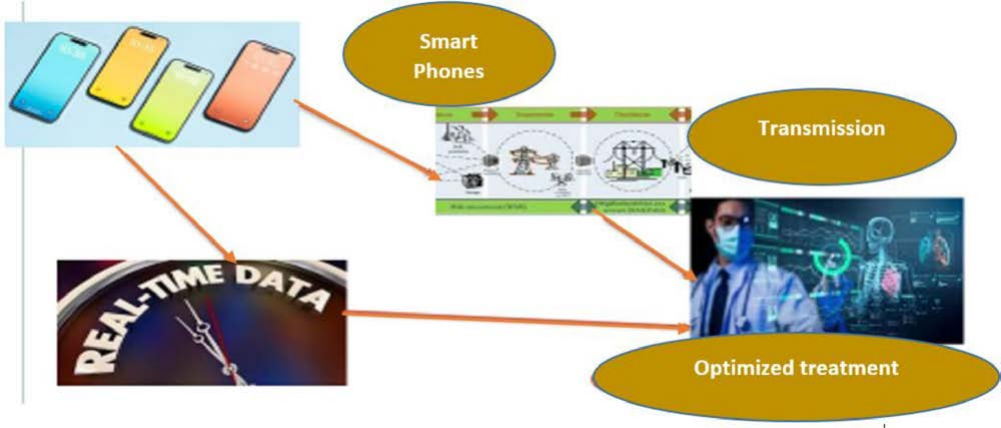
The adoption of mHealth technologies for real-time data collection and transmission.

Mobile health (mHealth) technologies offer significant potential for the alteration of healthcare providing through instantaneous data collection and reporting.^[56]^ Wearable sensors, smartphone apps, cloud computing, and wireless communication technologies make continuous monitoring, tailored interventions and improved patient outcomes feasible as shown in Figure 5. Despite of the fact that there are many challenges, the practice of innovation and implementation mHealth solutions would produce the future of healthcare, which is more reachable, efficient, and patient-centric^.[60]^

### 2.12. The unification of genomic sequencing with advanced analytics for quick identification and tracking of pathogen

Infectious diseases are major public health threats and therefore, rapid pathogen identification and tracking are essential in controlling their effects.^[60]^ Culture-based methods and serological assays are the classic approaches to pathogen identification and characterization but are rather slow, expensive, and restricted in providing detailed genetic information.^[64]^ Genomic sequencing, by contrast, is a useful methodology for the comprehensive analysis of microbial genomes, facilitating accurate pathogen identification, and details of their evolutionary dynamics.^[84]^ The era of next-generation sequencing (NGS) technologies has democratized genomic sequencing which enables high-throughput and cost-effective analysis on many pathogen genomes.^[85]^ Genomic sequencing in conjunction with progress in bioinformatics tools and computational resources has provided an opportunity for fast and precise pathogen detection in on both clinical samples, environmental sources, and surveillance data^.[86]^ In addition, the adoption of high-level analytics including machine learning algorithms and network analysis methods has improved the usefulness of genomic data in pathogen identification and tracking.

This review outlines the synthesis of the genomic sequencing and advanced analytics for rapid identification and tracking of pathogens. It also covers the principles and methodologies of genomic sequencing, demonstrate the role of advanced analytics in improving pathogen analysis in terms of speed and accuracy, and provide a number of applications of this integrated approach in public health surveillance, outbreak investigation, and clinical diagnostics^.[85]^

### 2.13. Genomic sequencing technologies

Sequencing of the genome includes identification of nucleotide order of an organism’s genome, which provides a number of information about its genetic make-up, evolutionary past, and functional properties^.[84]^ The sequencing technologies of the past few decades have made significant progress, which resulted in the emergence of NGS platforms that provide fast and high-throughput sequencing at reduced costs.^[85]^ Key NGS technologies are Illumina sequencing, Ion Torrent sequencing, and Oxford Nanopore sequencing and they all have their strengths and weaknesses. Illumina sequencing, which utilizes reversible terminator chemistry, is known for its high accuracy and high throughputs and is therefore well-established in whole genome sequencing and targeted sequencing applications^.[85]^ Ion Torrent sequencing with semiconductor sequencing technology is fast with a simpler workflow but is less accurate in comparison to Illumina sequencing. Oxford Nanopore sequencing, which is nanopore technology-based, allows real-time sequencing of long DNA fragments with low sample preparation needs and high error rates.^[86]^ The sequencing platform that is good for a study includes the sequencing depth, sample type, and resources available. NGS technologies have transformed pathogen genomics, regardless of the platform, allowing for the comprehensive analysis of microbial genomes at an unparalleled scale and resolution.^[85]^ Genomic sequencing data analysis needs powerful bioinformatics pipelines that consist of a variety of computational tools and algorithms for data preprocessing, quality control, variant calling, and phylogenetic analysis^.[86]^ Pipelines are a must-have for turning raw sequencing reads into interpretable information about pathogen genomes, which allows for a quick and accurate identification of microbial species, antimicrobial resistance genes, and virulence factors.^[85]^ For pathogen analysis, bioinformatics tools that are commonly used includes reading aligners (e.g., g., GATK, Freebayes, Platypus) and annotation tools (e.g., Annovar, Funcotator). g. the common software include GenomeEBrowser, SNP caller (e.g., RAxML, BEAST). Moreover, pathogen-specific pipelines designed for distinct purposes, such as outbreak investigation or antimicrobial resistance surveillance, have been created to simplify the analysis process and improve the interpretability of genomic information.^[85]^

### 2.14. Pathogen identification and tracking advanced analytics

Advanced analytics, beside bioinformatics pipelines, are also important in the derivation of meaningful insights from genomic sequencing data and for enabling rapid pathogen identification and tracking.^[87]^ Notably machine learning algorithms are promising in the prediction of antimicrobial resistance profiles, detection of transmission clusters, and species classification based on genomic signatures.^[87]^ The supervised machine learning methods endeavored to have been applied are support vector machines and random forest, and they are trained on the annotated genomic data to predict the phenotypic traits of interest from genomic sequences, such as drug resistance or virulence potential.^[88]^ The network analysis methods can offer another way of investigating the complicated interrelationships between pathogens, hosts, and environmental factors.^[89]^ Pathogen genomes as nodes and their relations as edges are represented by the network analysis allowing visualization and quantification of transmission dynamics, genetic diversity, and community structure within microbial populations.^[90]^

### 2.15. Public health surveillance and outbreak investigation applications

The application of genomic sequencing with advanced analytics has transformed public health surveillance and outbreak investigation by giving prompt and actionable information on the transmission dynamics and epidemiological attributes of infectious diseases.^[91]^ Most notably, this method has been essential in the detection and control of outbreaks of bacterial pathogens, such as *Salmonella, Escherichia coli*, and *Mycobacterium tuberculosis*.^[92]^ Sequencing the genomes of clinical isolates and comparing them to reference databases allows public health agencies to promptly identify the pathogen associated with an outbreak, track its source, and introduce targeted control measures aimed at limiting further spread.^[93]^ Also, genomic epidemiology enables discrimination of the closely related strains in outbreak clusters and identification of transmission chains and super-spreading events^.[94]^

### 2.16. Applications in clinical diagnostics

Apart from being useful in public health surveillance and outbreak investigation, genomic sequencing offers an enormous potential for enhancing clinical diagnostics and personalized medicine.^[95]^ The rapid sequencing and analysis of pathogen genomes directly from clinical samples allow clinicians to act upon informed treatment decisions, optimize antimicrobial therapy, and monitor in real-time the emergence of drug resistance.^[96]^ For instance, in the scenario of hospital-acquired infections, genomic sequencing can reveal the origin of transmission and direct the infection control measures aimed at avoiding further spreading.^[95]^ Also, in the area of antimicrobial stewardship, genomic analysis identifies appropriate antibiotics based on the genetic makeup of the infecting pathogen hence decreasing the risk of treatment failures and the spread of resistant strains^.[96]^

### 2.17. Challenges and future directions

Although it has the potential to be transformative, the integration of genomic sequencing and advanced analytics for rapid pathogen identification and tracking has a number of challenges and limitations.^[74]^ These comprise the issues of standardized protocols and quality control parameters, extracting complex genomic data in the clinical context, and the moral and regulatory issues regarding data sharing and privacy^.[75]^

### 2.18. Enhancing conflict zone monitoring

Conflicts zone are areas of instability, violence, and humanitarian crises and are thus very hard to monitor and to monitor and carry out surveillance.^[23]^ These environments often make traditional surveillance methods ineffective since access is restricted, the terrain may be prohibitive, and the presence of human intelligence is risky, among other things^.[34]^ Advanced surveillance technologies like UAVs, remote sensing, AI, and Big Data Analytics have emerged as a potential alternative in recent years for addressing these challenges.^[35]^ In this paper, we critically reviewed the successful implementation of these technologies in conflict zones and their influence on conflict management and resolution as shown in Table 2.

**Table 2:** Published literature and case studies on technological intervention in conflict-affected zones.

Title	Authors	Year	Technology	Application	Summary	Challenges highlighted
“Unmanned Aerial Vehicles (UAVs) for Medical Supply Delivery in Conflict Zones”	^[97,98]^	2020	UAVs	Medical Supply Delivery	Case study on the use of UAVs for delivering medical supplies to remote and conflict-affected areas.	Security risks, airspace restrictions, operational reliability.
“Mobile Health Clinics in Conflict Zones: A Review”	^[99,100]^	2019	Mobile Health Clinics	Healthcare Services	Review of mobile health clinics providing essential healthcare in conflict-affected areas.	Logistics, security, staffing.
“Drones for Humanitarian Aid: Potential and Challenges”	^[52,101]^	2021	UAVs	Humanitarian Aid	Examination of drones used for delivering aid and conducting surveillance in conflict zones.	Technical reliability, legal and ethical concerns, acceptance by local communities.
“Telemedicine in War Zones: Bridging the Gap”	^[102,103]^	2018	Telemedicine	Healthcare Services	Analysis of telemedicine systems deployed in conflict zones to provide remote healthcare.	Connectivity issues, data security, trust in technology.
“Solar-Powered Water Purification Systems for Conflict Areas”	^[104,105]^	2017	Solar Technology	Clean Water Supply	Case study on solar-powered systems providing clean water in conflict zones.	Maintenance, theft, initial setup costs.
“GIS Technology for Conflict and Post-Conflict Recovery”	^[106,107]^	2019	GIS	Disaster Management	Use of GIS technology to manage and coordinate relief efforts in conflict-affected areas.	Data accuracy, training requirements, infrastructure damage.
“Blockchain for Humanitarian Aid in Conflict Zones”	^[108,109]^	2020	Blockchain	Aid Distribution	Exploring the use of blockchain to ensure transparent and efficient aid distribution.	Technical complexity, acceptance by aid organizations, security risks.
“Satellite Imagery for Monitoring Conflict and Humanitarian Crises”	^[110,111]^	2022	Satellite Imagery	Surveillance and Assessment	Use of satellite imagery to monitor and assess humanitarian crises in conflict zones.	Data interpretation, timely updates, high costs.

This table highlights various technological interventions, their applications, summaries of their usage, and the specific challenges encountered in conflict-affected zones.

### 2.19. Case studies and examples

*UAVs for surveillance*: Surveillance in conflict zones has been revolutionized by UAVs, also known as drones.^[50]^ During the conflict in Syria, various actors, like the government forces and non-state armed groups, have been implementing the use of small UAVs equipped with high-resolution cameras and infrared sensors in gathering intelligence, tracking enemy movements, and conduct targeted strikes.^[52]^ UAVs have provided the capability of operational surveillance in real time across wide and distant areas of interest of ground units, enabling tactical advantage for armed forces.^[53]^

*Remote sensing for environmental monitoring*: Remote sensing technologies (satellite imagery and aerial photography) proved to be very useful in monitoring environmental changes and exploitation of resources in conflict zones^.[45]^ The Democratic Republic of the Congo, satellite imagery is being applied in monitoring deforestation, illegal mining activities, as well as movement of the armed groups in the remote areas.^[112]^ The interpretation of satellite data allows researchers and humanitarian organizations to locate the areas that are in danger of conflict escalation and to select the areas that need efforts of intervention.^[50]^

*AI and predictive analytics*: AI and machine learning algorithms offer hope to analyze huge volumes of data, to find patterns and to predict conflict dynamics and potential hotspots before the violence happens.^[70]^ In South Sudan, researches have been working on the predictive models, which take into consideration such factors as socio-economic, political and environmental, and allow to forecast the possibility of violence in the separate regions^.[113]^ Using AI-driven analytics, policymakers and peacekeeping forces can preposition resources and take preventive measures to reduce conflict risks.

*Challenges and ethical considerations*: Advanced surveillance technologies have a lot of benefits in the monitoring of the conflict zones, but their use does not conveniently occur due to the challenges and ethical concerns.^[114]^ Key challenges include:

*Privacy and civil liberties*: Surveillance technologies are used presumptively and the question of privacy rights and civil liberties is raised especially in authoritarian regimes where these technologies may be utilized for political repression and human rights violations.^[80]^

*Data security and cyber threats*: Sensitive surveillance data collection and storage create risks of unauthorized use, data leaks, and cyber-attacks that can threaten operational security and put people at risk.^[82]^

*Bias and accuracy*: The AI algorithms applied in predictive analytics may develop biases and problems, causing wrong conclusions and erroneous decision-making. Besides, too much dependent on technology-based responses may fail to take into account contextual subtleties and local dynamics which minimizes the effectiveness of conflict prevention strategies.^[87]^

Advanced surveillance systems have come in handy in monitoring conflict zones, increasing situational awareness, and facilitating conflict resolution initiatives.^[88]^ The paper has shown through case studies and examples, the different applications and advantages of UAVs, remote sensing, AI, and predictive analytics in conflict environments.^[92]^ Yet, the effective utilization of these technologies demands a balanced approach to ethical, legal, and operational issues. In conclusion, interdisciplinary cooperation among researchers, policy makers, and technology developers is crucial for maximizing the benefits of advanced surveillance technologies in promoting peace and security in conflict affected areas.^[113]^

### 2.20. Challenges in flying drones in conflict-affected zones

It is important to mention certain challenges operating drones within conflict-affected areas characterize. Although UAV technology is well-established in defense applications, its use in healthcare, particularly in such volatile environments, introduces unique obstacles.^[23,34]^

### 2.21. Security concerns

*Hostile forces*: Drones might be attacked and destroyed by the enemy or alternatively, captured by an enemy power. A malicious party can thus cause a significant loss of expensive equipment and possibly gain access to sensitive technology.^[35,45]^

*Misidentification*: But possibly the most serious is the hazard of drones being mistaken for military equipment, resulting in strikes from both local and international forces.^[23,34]^

### 2.22. Operational challenges

*Jamming and hacking*: It is a well-known fact that areas which are involved in armed conflicts could be experiencing pretty high levels of electromagnetic activity and attempts at jamming and hacking. This can interfere with the link between the control center and the drone or with its navigation system, resulting in unsuccessful or crashed missions.^[45]^

*No-fly zones*: Some of the conflict problems include the fact that most conflict regions have closed the airspace because of military activities, which limit permission to fly or navigate around no-fly zones^.[45]^

### 2.23. Environmental factors

*Terrain*: However, it can be hampered if the environment is rugged, mountainous or urban terrain which makes navigation or operational efficiency prohibitive.^[50]^

*Weather conditions*: Storms are also evident in most conflict regions and represent a challenge to stability as well as a reduction in battery strength and flight performance.^[52]^

### 2.24. Logistical issues

*Deployment and maintenance*: In establishing and maintaining the operations of the drones, one needs a safe place to launch control and recover the drones, steady source of supplies for the spare parts and fuel, and personnel skills, factors difficult to secure in conflicted regions^.[53]^

*Recovery of drones*: When the drone is down in a hostile area or an area that cannot be accessed easily the job of retrieving them become a very risky affair^.[45]^

### 2.25. Ethical and legal considerations

*Privacy concerns*: Security and healthcare are great areas of interest when it comes to the use of drones especially for surveillance and delivery of services but there are concerns with regards to privacy particularly of individuals in conflict prone areas^.[23,35]^

*Legal restrictions*: There are numerous international laws and so many local off air laws that may limited the use of drone especially in sovereign airspace or near military sensitive facilities.^[45]^

### 2.26. Public perception and acceptance

*Trust issues*: The people on the ground may have some reactions to drones, they may find them being an invading vehicle rather uncomfortable and therefore rebel against it.^[50]^

*Community engagement*: The potential to communicate and involve people directly exerts pressure on the authorities and citizens, which is often difficult in conflict-sensitive environments.^[53]^

### 2.27. Ethical and legal considerations

Surveillance technologies which include CCTV cameras, drones, facial recognition systems, biometric identification tools are being found to be deployed in conflict zones across the globe.^[115]^ Despite that these technologies have many virtues for the enhancement of security, intelligence gathering, and law enforcement, they represent significant ethical problems which include privacy, data ownership, and informed consent.^[116]^ The practice of surveillance technologies in such conflict situations as are marked by high tension, human right abuses, and authoritarian regimes occurs within an ethical dilemma.^[117]^ The review brings up the ethical consequences of the use of surveillance technologies in such environments, for the purpose of awakening serious thoughts and the input of policy debates.

*Privacy concerns*: Privacy is based on moral and legal international instruments as an element of human right. Yet, the surveillance technologies employed in conflict situations are a danger to the private rights of an individual; a person cannot retain anonymity and individual sovereignty under the systematic surveillance and gathering of information.^[115]^ Mass surveillance practices such as undirected data collection and dragnet surveillance lead to erosion of privacy shield and state surveillance machinery becoming an operation.^[116]^ Furthermore, secrecy and irresponsibility towards surveillance programs exacerbate the privacy concepts and create a suspicious attitude in the people, who are under surveillance^.[115]^ However, the threat of data breach, misuse of personal information and unauthorized access contributes only to privacy risks, which is an issue of ethical warrant for the use of invasive surveillance methods.^[117]^

*Data ownership*: Data ownership is central to discussions of surveillance technologies in situations where there is conflict.^[115]^ While states and security agencies generally assert their ownership rights in data collected in the public sphere, the question is who is the rightful owner of personal data and metadata created by individuals? In the absence of clear legal and consent frameworks, people may lose control over their data and suffer harm.^[116]^ Besides, the commercial character of surveillance data monetization by the non-state actors introduces a commercial factor which complicates matters of ownership and liability.^[115]^ Ethical concerns also demand a clear delineation of ownership rights of the data and the provision of tools to enable individuals to manage their personal details and control the use of data upon them.^[116]^

*Informed consent*: The principle of the informed consent is a cornerstone of ethical research and data collection in all areas, though, in the conditions of the surveillance technologies in conflict settings, it is confronted by many challenges.^[114]^ Coercive surroundings, all-pervading teaching, and inequalities render the affirmation authentic to data collection and data use. Furthermore, the surveillance issue can make the victims of refugees, internal displacement victims, and detainees viability higher and limit the consent^.[115]^ Informed consent procedures are ethical matters and transparency, voluntary and culturally appropriate, as well as, involvement, feedback, and complaint mechanisms are involved.^[115]^ However, preserving ethical approval in conflicting environment comes in refinishing the sensitive issues of who and how to resolve the person autonomy and agency in such context.

Surveillance technologies in conflict areas raise questions of ethical nature which generally concern privacy, data ownership rights and informed consent.^[88]^ For this purposes, there is a need for a proactive approach ensuring the rights protection of citizens, responsible data governance, and ethical usage of surveillance technologies, but the technological progress is outrunning the development of regulatory frameworks and ethical norms.^[89]^ Transparent governance structures, stakeholder participation, and human rights impact assessment are important to the ethics risks and help observation practice in compliance with democratic values and international human rights standards.^[89]^ Finally, ethical discussions of surveillance technologies in conflict settings need to be balanced and reconcile the security objectives with human privacy, dignity, and autonomy.

### 2.28. Surveillance technologies and humanitarian assistance in health emergencies

Considering health crises—pandemics, natural disasters and conflicts—integration of surveillance Technologies with humanitarian aid efforts offers the great perspective to strengthen preparedness, response, and recovery strategies.^[80]^ Yet, this convergence introduces both ethical, legal and operational issues relating to accountability, transparency, and potential abuse.^[83]^ This review integrates the latest literature in exploring the relevance of coordination between technological innovations and humanitarian aid, with attention to how accountability is maintained, surveillance risks are addressed, and resource allocations are optimized through capacity building initiatives.^[84]^

*Ensuring accountability and transparency*: The handling and processing of sensitive health information in humanitarian settings require sound accountability systems to protect the rights of individuals and reduce the chances of the data being misused.^[85]^ Data governance transparency, which involves clear rules as to data collection, storage, and sharing, is critical in creating trust and accountability among stakeholders. Additionally, surveillance decisions should be based on ethical frameworks like informed consent, data anonymization, and data minimization that are meant to protect privacy and autonomy rights.^[86]^ Creating frameworks of an autonomous monitoring, public participation, and complaint redress can significantly improve accountability and transparency in the employment of surveillance tools for humanitarian goals.^[85]^

*Addressing potential misuse*: The risk of surveillance technologies being abused by either state actors or non-state actors for the purposes of surveillance emphasizes the need for strict checks and balances and regulatory systems^.[87]^ The development of legal and ethical guidelines is essential to guard against unauthorized access, data breaches, and forms of discrimination that violate individual rights.^[88]^ Risk assessment, impact evaluation, and human rights impact assessment mechanisms can enable the identification and mitigation of risks caused by misuse of technology. Further, such collaboration aids in ethical innovation and accountability of surveillance technologies in humanitarian contexts^.[89]^

*Integration with humanitarian aid delivery*: Surveillance technologies with humanitarian aid delivery integration create the possibilities of resource allocation optimization, improvement in the situational awareness as well as the response efficiency.^[89]^ Advanced surveillance data, such as near real-time disease surveillance, population movement patterns, and health infrastructure mapping, provides evidence-based decision making and prioritization of interventions in crises.^[90]^ Through technology-based approaches, humanitarian organizations can improve their capability to provide timely and focused aid to affected communities, therefore reducing morbidity and mortality rates among health emergencies.^[91]^

*Capacity-building initiatives*: Capacity development programs are very important to give power to the local healthcare workers as well as to the aid personnel to use surveillance technologies in the time of crisis.^[90]^ Training programs, workshops and knowledge-sharing platforms could enable the frontline responders to have the skills and knowledge to gather, analyze and interpret surveillance data. In addition, collaboration of technology providers, educational institutions and local actors enables joint design solutions that correspond with the local requirement of victimized communities.^[92]^ Investing in development of human capacitance, humanitarian organizations enable to improve local resilience, develop innovations, and achieve long-term impact in health crisis response efforts.^[93]^

The coming together of surveillance technology with humanitarian aid gives opportunities that have never been seen before concerning response capacity and health improvement in crisis settings.^[94]^ Nevertheless, the full efficiency of such integration can only be achieved through substantial work aimed at ensuring accountability, transparency, and ethical treatment of sensitive health information.^[95]^ Through provision of integration between technology innovations and traditional humanitarian frameworks, stakeholders can ensure resource allocation, improve response capacity, and reduce risks of surveillance technologies.^[95]^ Capacity-building initiatives are central to enabling local actors, and as such, promoting sustainable solutions that put the welfare and dignity of affected populations first.^[88]^ Technology and humanitarian aid should be integrated at the core of the responses to the health crises that we are navigating in an ever more connected world, where the complexity of the challenges can only be addressed with collaboration and innovation, in order to build resilient communities and protect public health.^[89]^

### 2.29. Using technology in conflict areas for improved infectious disease response

Conflict settings make the burden of infectious diseases worse; they create more health issues and make responses more complicated^.[90]^ Nevertheless, technology offers some of the opportunities to strengthen infectious disease response in the setting. This review sets out to determine policy recommendations that are required to support the integration of technology into infectious disease response strategies in conflict areas.^[91]^

*Surveillance and early warning systems*: Surveillance is essential in the prompt identification and response to outbreaks of infectious diseases.^[92]^ Governments, international institutions, and nongovernmental organizations should invest in the creation and practical use of systems of real-time monitoring on technology.^[94]^ This is through use of mobile health (mHealth) applications, geographic information systems and remote sensing technologies to track disease dynamics and pinpoint hotspots in conflict zones^.[70]^

*Diagnostic tools and telemedicine*: Many conflict settings experience a challenge of limited availability of rapid and accurate diagnostic tools thus, disabling disease identification and management.^[67]^ Policy interventions should concentrate on the deployment of point-of-care diagnostic systems such as the rapid diagnostic tests and portable molecular assays that will enable timely diagnosis of the infectious diseases in rural and resource-poor settings.^[68]^ Furthermore, telemedicine solutions enable distant consultations and help healthcare workers in war areas by offering virtual training and mentoring sessions.^[69]^

*Treatment and drug delivery*: Access to basic medicines and health care services is a thing to achieve in conflict-affected zones.^[80]^ Governments and international organizations should partner with pharmaceutical companies and humanitarian organizations in the development of the innovative drug delivery modalities that include UAVs, and blockchain-powered supply chain management systems to deliver medications safely to hard-to-reach regions.^[87]^ In addition, telemedicine can be utilized for remote monitoring of patients and medication compliance which will enhance the treatment outcomes of patients in conflict settings.

*Community engagement and behavior change*: In conflict zones, community involvement is critical for infectious disease interventions to be successful. NGOs are important in trust building with the local communities and behavior change initiatives.^[89]^ In this regard, policy recommendations should focus on creating culturally appropriate communicational tactics and community-based participative approaches that will use digital platforms and social media channels to spread health information and promote compliance with preventive measures.

*Capacity building and partnerships*: Capacity building activities are necessary for the reinforcement of local healthcare systems and improvement of technological capacities of the conflict-affected areas.^[90]^ Investments should be focused on training programs for healthcare workers, technicians, and community health workers that will enable them to use technology for infectious disease response in accordance with governments, international organizations, and NGOs^.[93]^ In addition, developing alliances between public and private sectors, academic organizations, and local participants will enable contextual co-creation and use of technology solutions.

Technology integration into infectious disease response strategies offers a change opportunity for addressing health disparities and outcomes improvement in conflict environments^.[95]^ On the other hand, successful implementation demands integrated policy interventions from the policy-makers, international agencies, and NGOs^.[96]^ Using the policy recommendations given in this review, the stakeholders can leverage the strength of technology in improving infectious disease surveillance, diagnosis, treatment and prevention in conflict–affected areas as well as saving lives and controlling epidemic impact in vulnerable populations.

### 2.30. The role of telemedicine, drugs and diagnostics delivery by drones in the conflict zone

#### 2.30.1. Telemedicine

*Role*: Telemedicine refers to the process of offering healthcare services by using ICTs to reach the patients without direct physical contact as shown in Table 3. It encompasses video consultation, remote diagnosis, electronic prescriptions, and nearly continuous patient monitoring^.[23,34,35]^

**Table 3:** The role of telemedicine and drone delivery in conflict zones.

Intervention	Role	Benefits	Challenges	Case studies	Authors	Year
Telemedicine	Provides remote healthcare services through digital platforms, enabling access to medical consultations, diagnostics, and follow-ups.	Extends healthcare access to remote and conflict-affected areas. Reduces the need for physical presence of healthcare professionals. Facilitates continuous care despite infrastructure damage.	Connectivity issues in conflict zones. Data security and patient privacy concerns. Limited local capacity to operate and maintain technology.	Telemedicine in War Zones: Bridging the Gap	^[23,34]^	2018
Drone Delivery (Drugs & Diagnostics)	Utilizes unmanned aerial vehicles (UAVs) to deliver medical supplies, including drugs and diagnostics, to hard-to-reach areas.	Rapid and efficient delivery of essential medical supplies. By passes damaged or blocked infrastructure. Reduces risk to humanitarian workers by minimizing the need for ground transportation in dangerous areas.	Security risks, including potential targeting by armed groups. Airspace restrictions and regulatory barriers. Technical reliability and operational challenges in hostile environments.	Unmanned Aerial Vehicles (UAVs) for Medical Supply Delivery in Conflict Zones	^[34,45]^	2020

*Benefits*: Presumably, telemedicine can greatly reduce the difficulties that are associated with the destruction of such healthcare systems in conflict-affected areas. It empowers doctors and nurses to care for patients without physically examining them, to monitor chronic diseases and deliver immediate care when exacerbating symptoms arise.^[23]^ Also, telemedicine solutions can be used as the knowledge base and a method of assisting local healthcare specialists.^[34]^

*Challenges*: There are challenges that telemedicine faces when offered in conflict regions. Internet and communication networks are mostly very unreliable, and this remain the greatest challenge in attaining consistent connectivity.^[23]^ Patient data is also at risk and vulnerable to breaches especially in the unstable regions where data leak could be dangerous.^[34]^ In addition, the ability of the local provider to sustain and administer the telemedicine equipment could be weak, and this requires constant capacity enhancement.^[35]^

#### 2.30.2. Medical drones, or UAVs

*Role*: Medical drones, or UAVs, are employed in delivering medicines, vaccines, diagnostic equipment and specimens, as well as in transporting deliveries from and to distant or zones of armed conflicts. This method is especially used when the ordinary roads are not safe or navigable due to various reasons.^[34,45,50]^

*Benefits*: The first benefit that stems from the use of UAVs for delivery of medical supplies is the relatively fast and effective way of reaching the deserving populace.^[45]^ Drones can maneuver through the scarred landscape and avoid areas of destruction, thus, making sure that food and other essentials are promptly delivered.^[50]^ This alleviates concerns that are typically associated with a humanitarian organization in terms of transport needs and risks, which face personnel require passing through dangerous areas to deliver aid.^[52]^

*Challenges*: Nevertheless, the use of drones has its hurdles in the theaters of conflict. Security risks are critical, as the drones developed could be attacked or hijacked by hostile actors.^[52]^ However, there are also some legal aspects of airspace, which can be claimed by different authorities and turned into a kind of a conflict.^[50,52]^ Paramount challenges that arise in technical aspect include aspects like ensuring that the drone is reliable and or handling some technical hitches that may arise especially in a hostile environment where technical support may not be easily accessible.^[53]^

### 2.31. Beyond visual line of sight (BVLOS) drone delivery in no network zones

Over and above visual line of sight delivery using drones has become an innovative solution in the transport and delivery segment that has the capability to deliver consignments to previously unreachable regions that are uninhabited or have poor infrastructure.^[118]^ BVLOS enables drones’ operations beyond the range of the operator’s sight exposure; hence, it offers a more extended viewing distance.^[118]^ This capability is especially desirable in no network zones where other communication systems are nearly nonexistent or are not efficient at all.^[119]^

### 2.32. Technological foundations

BVLOS drone operations use more complex communication and navigational facilities than other operations.^[120]^ Satellite communications, radio frequency links, and onboard self-navigation systems that are engineered by global positioning system along with inertial measurement units are some of the standard methods^.[120]^ Additional and new forms of AI and machine learning subsequently improve the flight dynamics, empowering the drones to maneuver past obstructions independently of a person’s immediate control^.[118]^

### 2.33. Regulatory landscape

To this end, there are still no concrete and standard regulations that govern BVLOS operations, with the various countries in the world having put in place dendritic standards as well as guidelines.^[121]^ Where BVLOS flights are involved, in the United States for instance, Special Waivers may be granted under the Federal Aviation Administration but the emphasis is on safety and risk assessment. Globally, the International Civil Aviation Organization is striving hard in an attempt to come up with more standardized regulations in an effort to enable seamless BVLOS operations^.[121]^ These regulations often require that stringent safety features be put in place like back up communication-links, and safety measures to be taken in the event that the link is lost.^[119]^ The following is a list of specific areas that are challenging to operate in when there is no network connection. In no network zones, the operation of BVLOS drones poses certain concerns that other categories of drones do not present.^[120]^ The first problem is weak communication provision, especially the hardware on which timely controlling and monitoring are based.^[121]^ Satellite communication systems is a viable solution the only is that it covers a larger area but it may come at a higher price and it may also have a lag.^[122]^ Self-navigation and self-protection systems are crucial factors that should be considered in mitigating risks arising from the loss of communication links, either periodically or permanently.^[120]^

### 2.34. Case studies and evidence

The issues of BVLOS drone delivery in no network zone have been sparsely proven by few pilot projects and case studies.^[123]^ For instance, a similar medical drone delivery named Zipline has managed to use BVLOS drones to deliver medical supplies in the rural areas of Rwanda and Ghana.^[119]^ These projects have involved demonstration of proportional short delivery time and improved accessibility especially in emergency medical transport.^[120]^ These demonstrations prove that BVLOS technology can open up new opportunities for logistics when other means are unavailable in remote regions.^[121]^

### 2.35. Technological innovations

The BVLOS technology is being developed day by day and with modern features and functions being added to the drones.^[118]^ The advancement in battery technology and efficient flight modes is helping drones to configure longer flying time beyond their previous operational limits.^[119]^ Also, there exists advancement in the use of AI and machine learning for improving the autonomy of drones, that is, its capability to decide at any given moment on what action to undertake in a specific environment which has been developed to offer real-time decisions.^[120]^ Wireless mesh network technology in which drones are capable of relaying information to other drones in nearby areas to extend coverage are also being proposed to address the no network zone problem.^[121]^

### 2.36. Future directions

The future of BVLOS drone delivery in no network zones depends on still advancing technology and favorable regulations.^[122]^ However, there are still open issues that require research in order to solve them, like to enhance satellite communication reliability, and to lessen the costs associated with it.^[123]^ Recommendations on the policy front include placing pressure on governments to adopt global standards for BVLOS operations across the world and organizing more capital to bring in more BVLOS structure for drones.^[123]^

## 3. Conclusion

Infectious disease outbreaks in conflict zones pose significant challenges to public health and humanitarian efforts, requiring new methods of surveillance, diagnosis, and response. This narrative review has illuminated the synergistic relations between conflict dynamics, healthcare infrastructure, and technological innovations in determining infectious disease outcomes in such settings. The destruction induced by conflicts not only devastates the physical infrastructure but also undermines healthcare systems, making the situations even more vulnerable and difficult to access essentials healthcare services. Migration of population makes disease surveillance and response more difficult and calls for get used to strategies to meet changing population dynamics and ensure uniform healthcare. Advancements in technological innovation including in surveillance, mobile health (mHealth) technologies, genomic sequencing, and surveillance technologies present some hope for the improvement of infectious disease response in conflict zones. Such innovations promote live surveillance, remote diagnostics, and predictive analytics, which facilitate the early detection of outbreaks and timely response action. Nonetheless, technology has some integration challenges in infectious disease response strategies in warfare areas. In maximizing the impact of technology interventions while safeguarding individual rights and promoting equity, healthcare workers and humanitarian agencies security risks, ethical considerations related to data privacy and surveillance, and resource constraints should be well managed. The governments and international institutions should allocate resources for the development and implementation of strong epidemiological surveillance systems in conflict-ridden territories. This consists of the use of modern technologies like UAVs, AI, BVLOS, and big data analytics to improve real-time monitoring and early warning systems. Diagnosis capacity in conflict zones should be improved by the use of portable diagnostic tools, laboratory infrastructure development, and training of healthcare personnel. This also involves the use of genomic sequencing technologies to enable quick identification as well as tracking of pathogens. Capacity development programs should target healthcare workers and humanitarian responders to be trained in using technology based tools and strategies for infectious disease surveillance, diagnosis, and response. This involves delivering security awareness training to manage hazards frontline responders are confronted with. Governments, international organizations, academia, and the private sector should work together to create and apply technology-driven infectious disease response strategies in areas affected by conflicts. This involves generation of collaboration agreements for the purpose of data sharing, technology transfer and innovation. Ethical governance frameworks should be set up to govern the ethical usage of technology in conflict settings, which guarantees data privacy, security and transparency. This involves carrying out human rights impact assessments and stakeholder consultations to avoid possible threats and protect the rights of the people. These policy recommendations when implemented would enable stakeholders to maximize the potential of technology in response to infectious diseases in conflict zones to reduce human suffering and enhance global health security. Nevertheless, long-term political will, financial input, and multi-disciplinary partnership will be critical to address the difficulties that these environments present and to realize a lasting effect.

## Author contributions

**Conceptualization:** Okechukwu Paul-Chima Ugwu, Esther Ugo Alum.

**Data curation:** Okechukwu Paul-Chima Ugwu, Esther Ugo Alum, Jovita Nnenna Ugwu, Val Hyginus Udoka Eze, Chinyere N Ugwu, Fabian C Ogenyi, Michael Ben Okon.

**Investigation:** Ugwu Chinyere N, Fabian C Ogenyi, Okon Michael Ben.

**Methodology:** Okechukwu Paul-Chima Ugwu, Jovita Nnenna Ugwu, Val Hyginus Udoka Eze.

**Supervision:** Val Hyginus Udoka Eze, Fabian C Ogenyi.

**Validation:** Val Hyginus Udoka Eze, Chinyere N Ugwu.

**Visualization:** Okechukwu Paul-Chima Ugwu, Val Hyginus Udoka Eze, Michael Ben Okon.

**Writing – original draft:** Okechukwu Paul-Chima Ugwu, Esther Ugo Alum, Jovita Nnenna Ugwu, Chinyere N Ugwu, Fabian C Ogenyi, Michael Ben Okon.

**Writing – review & editing:** Okechukwu Paul-Chima Ugwu, Esther Ugo Alum, Jovita Nnenna Ugwu.
